# What Is Ailing Oncology Clinical Trials? Can We Fix Them?

**DOI:** 10.3390/curroncol31070275

**Published:** 2024-06-28

**Authors:** Abhenil Mittal, Sara Moore, Vishal Navani, Di Maria Jiang, David J. Stewart, Geoffrey Liu, Paul Wheatley-Price

**Affiliations:** 1 North East Cancer Center, Health Sciences North, Northern Ontario School of Medicine (NOSM U), Sudbury, ON P3E5J1, Canada; amittal@hsnsudbury.ca; 2 Department of Medicine, Division of Medical Oncology, The Ottawa Hospital Cancer Centre, University of Ottawa, Ottawa, ON K1H8L6, Canada; 3 Tom Baker Cancer Center, Alberta Health Services, Calgary, AB T2N4N2, Canada; 4 Cumming School of Medicine, University of Calgary, Calgary, AB T2N4N2, Canada; 5 Princess Margaret Cancer Centre, University Health Network, University of Toronto, Toronto, ON M5G2M9, Canadageoffrey.liu@uhn.ca (G.L.)

**Keywords:** clinical trials, barriers, eligibility, trial activation, trial conduct

## Abstract

Evidence from phase three clinical trials helps shape clinical practice. However, a very small minority of patients with cancer participate in clinical trials and many trials are not completed on time due to slow accrual. Issues with restrictive eligibility criteria can severely limit the patients who can access trials, without any convincing evidence that these restrictions impact patient safety. Similarly, regulatory, organizational, and institutional hurdles can delay trial activation, ultimately making some studies irrelevant. Additional issues during trial conduct (e.g., mandatory in-person visits, central confirmation of standard biomarkers, and inflexible drug dosage modification) contribute to making trials non-patient-centric. These real-life observations from experienced clinical trialists can seem nonsensical to investigators and patients alike, who are trying to bring effective drugs to patients with cancer. In this review, we delve into these issues in detail, and discuss potential solutions to make clinical trials more accessible to patients.

## 1. Introduction

Case examples:

1. A patient with early triple-negative breast cancer on a clinical trial for circulating tumor DNA (ctDNA)-based screening after curative therapy tests positive and has a computed tomography (CT) scan showing metastatic disease. The physician wants to enroll her on a first line trial for metastatic disease; however, she is not eligible as the scan happened 350 days after finishing her curative treatment, rather than after >365 days. Does 15 days make a clinical difference?
2. A patient with metastatic castrate-resistant prostate cancer on a study drug has clinical progression (a significant increase in pain, requiring the initiation of opioid analgesia), along with a consecutive increase in prostate-specific antigen (PSA). CT and bone scan imaging show new progressive bone lesions corresponding to the site of pain; however, confirmed radiographic progression requires repeat imaging in eight weeks, as per the Prostate Cancer Working Group Criteria. The trial allows cross-over; however, only in cases where radiographic progression is confirmed centrally. In this clinical scenario, is it warranted to wait for confirmatory imaging and central review? Is unequivocal clinical progression not sufficient to allow cross-over?
3. A 58-year-old male has a non-contrast CT chest ordered by his family doctor because of a persistent cough. It shows a lung mass, and after comprehensive workup including PET scan and mediastinoscopy, he has a successful surgical resection for a stage 2a non-small-cell lung cancer. He is approached about an adjuvant systemic therapy trial, but because the initial CT chest had been performed without contrast, he is deemed ineligible. How do these criteria help either trial data, safety, or enrolment, and would more thoughtful protocol development have allowed many ‘ineligible’ patients to actually be eligible?

Clinical trials are the cornerstone for cancer research and providing high-quality practice recommendations. Early phase clinical trials are usually hypothesis generating, whereas phase III trials attempt to confirm the efficacy of a new treatment compared to an existing standard of care (SOC) [1]. The success of a clinical trial depends on recruiting and retaining an adequate number of representative patients to answer the question at hand, adherence to protocol during trial conduct, following good clinical practice (GCP) guidelines [2], and minimizing barriers to facilitate successful trial completion.

Previous studies have shown that only <5% of adult patients with cancer participate in cancer clinical trials [3,4,5]. Overall, >80% of clinical trials in the United States fail to finish on time, with >20% being delayed for >6 months [6,7,8]. Among the National Cancer Institute (NCI)-sponsored trials, around 40% of trials never reach completion, 20% are closed prematurely, with <50% meeting enrollment targets [9,10]. One study estimated a loss of approximately USD 1 million of low enrolling trials at a single academic institution in a single financial year [11]

Furthermore, certain subgroups including older adults with cancer [12,13], ethnic minority backgrounds [14,15,16,17,18], and certain comorbidities (e.g., HIV, renal disease, and brain metastasis) [19,20,21] are underrepresented in trials. Enrollment barriers can occur at the level of patients, physicians, and trials operating systems [10,22,23]. Investigators may have a limited awareness of ongoing clinical trials, time constraints in clinics, and few referrals for trials. Potential patients may lack health literacy to ask about clinical trials and have limited ability for travel and time requirements to participate in the study. The European Clinical Research Infrastructure Network (ECRIN) identified eight major barriers to RCTs. These included the following inadequacies: identification of a relevant research question; knowledge and understanding of clinical research; knowledge and understanding of clinical trials; funding; infrastructure and overly complex regulation; excessive, non-focused monitoring; as well as restrictive privacy and lack of transparency [24]. 

For clinicians and patients engaged in clinical research that are motivated to successfully bring new and effective treatments to the clinic, many barriers seem nonsensical, as they do not obviously impact patient safety or the ability of the study to answer the primary questions. Often, issues have been handed down from trial design to trial design without thoughtful modifications and evolution. In this review, we highlight key barriers to the successful conduct of clinical trials, from phases of trial design, trial activation, and conduct. We discuss potential strategies to help overcome these issues and factors for the consider of their implementation. In highlighting these barriers in this paper, we advocate for more flexibility and shared decision making between patients, clinicians, regulators, and trial sponsors, and hope to encourage more thoughtful trial design.

## 2. Section 1: Trial Eligibility and Design (Table 1)

Recent work has suggested that <50% of trials report a screen fail rate; when reported, ineligibility was the most common reason for screen failure [25]. The ASCO Friends for Cancer group have given several recommendations, but very few have been implemented. Most have only been converted to draft guidance by the FDA, rather than mandatory requirements for sponsors. Some of these are discussed below:

**Table 1:** Common issues with trial design and eligibility and suggested solutions.

Issues	Solutions
(a) Exclusion of patients with CNS metastasis (parenchymal and leptomeningeal)	Inclusion of patients with brain metastasis and LMD if not requiring urgent treatment, irrespective of time with stable metastasis
(b) Exclusion of patients with prior malignancy/controlled HIV	Should be included; exclusion criteria need to be clearly explained in the protocol
(c) Exclusion based on asymptomatic lab abnormalities	Avoid exclusion based on lab abnormalities as far as possible, especially if early phase data suggest safety. A cutoff based on an institutional lab is reasonable if early phase data are unavailable (bilirubin < upper limit of normal ULN and liver enzymes) (<3 times ULN, GFR 30–50 mL/min depending on drug). PI should have flexibility to adjudicate about eligibility if there is some deviation from lab value based on other patients’ characteristics [26]
(d) Exclusion based on asymptomatic QTc and ejection fraction abnormalities	Avoid exclusion based on ECG QTc criteria. Exclude only if arrythmias are clinically significant. Similarly, for ejection fraction, evaluation based on expected cardiotoxicity of the drug is essential rather than blanket exclusion
(e) Exclusion of older adults and those with ECOG PS ≥2	Allowing a subset of patients with higher ECOG PS or older age at the time of trial design

## 3. Central Nervous System Metastases 

Patients with brain metastasis or leptomeningeal disease (LMD) have been historically excluded from clinical trials. This issue is of particular importance in malignancies with a high propensity for CNS spread. A systematic review of ongoing trials in non-small-cell lung cancer (NSCLC) showed that only 40% of trials allowed patients with treated brain metastases, while 26% of trials allowed untreated brain metastasis, 14% excluded CNS metastasis all together, and 19% excluded LMD [27,28]. 

The exclusion of brain metastases from clinical trials is often unjustified [21]. While the blood–brain barrier (BBB) limits the penetration of some systemic agents into the normal central nervous system, it is largely disrupted by brain metastases. Many systemic agents can achieve high drug concentrations in brain tumors, leading to similar response rates for treatment-naïve brain metastases compared to extracerebral tumors. Effective therapies with established intra-cranial activity include ALK targeting tyrosine kinase inhibitors like brigatinib [29], alectinib/lorlatinib [30,31], and antibody–drug conjugates (ADCs) such as trastuzumab deruxtecan (T-DXd) [32]. 

The American Society of Clinical Oncology (ASCO) Friends for Cancer Brain Metastasis Research group put forward recommendations in 2016 for including most patients with brain metastasis in clinical trials if they are stable (symptomatic or asymptomatic). A reasonable time frame for stability of 4 weeks was discussed. However, there is no such period of stability requirement for other disease sites such as the liver, bone, or adrenal metastases, so why is this requirement necessary for brain metastases? Requiring time periods of CNS stability prior to inclusion on a trial may end up being a back-door way to obtain over-estimated efficacy outcomes. There have been recent studies of novel compounds exclusively in patients with brain metastases, many of those with symptomatic disease and vasogenic edema, outlining the potential for undertaking these studies in prospective clinical trials [32,33]. 

A pragmatic approach with regard to the use of anti-convulsant drugs was suggested, by increasing the use of non-enzyme-inducing anti-epileptic drugs such as levetiracetam, and only a minority of patients with brain metastases ever needed anticonvulsants [34].

The US Food and Drug Administration (FDA) has published guidance for industry advocating for the inclusion of patients with stable brain metastases in clinical trials, and requires a strong justification for their exclusion. Similar recommendations apply to patients with leptomeningeal disease (LMD). However, the practical implementation of these recommendations has lagged. In a study analyzing NCI-sponsored clinical trials between 2018 and 2020, only 15% explicitly implemented inclusion criteria for active brain metastasis [21]. Similarly, only 3% of trials (8/244) (all lung cancer studies) allowed the inclusion of patients with either asymptomatic or treated LMD, despite the growing number and efficacy of CNS-active drugs across cancer types [35]. 

## 4. Prior Malignancies and Treatments

Restrictive eligibility criteria around previous malignancies often exclude patients; however, many of these may not have bearing on the overall prognosis, safety, or efficacy of the investigational drug in question and can heighten age disparities in trials [36] (e.g., stage I breast cancer on surveillance). 

Similarly, not all exclusion criteria based on prior cancer treatments are justified. For instance, some trials for metastatic disease require patients who need palliative radiation to be taken off the study due to concerns of overlapping toxicity, even though there is no adequate supporting evidence.

## 5. Comorbidities

The ASCO Friends for Cancer group has also advocated for a broader eligibility for patients with well-controlled HIV in oncology trials if they are otherwise stable [37], with the support of the FDA [38]. With the current treatment landscape of HIV, and the almost normal life expectancy for treatment-compliant patients with normal CD4 counts [39,40], there is thought to be no additional risk with anti-cancer drugs that should hinder trial participation [37,41,42,43]. Most trials also exclude patients with serological evidence of Hepatitis B and Hepatitis C, given the risk of reactivation; however, with the availability of effective anti-viral drugs, the risks have decreased substantially and exclusion based on serological evidence alone should no longer be considered standard [38].

Patients with mild renal/hepatic/cardiac dysfunction may also be excluded because of arbitrary “cutoffs” rather than clinical relevance (usually >60 mL/min for GFR, >50% ejection fraction or <450 milliseconds for corrected QT interval for cardiac status, <2–3 times upper limit of normal (ULN) of liver enzymes, and bilirubin of <1.5 mg/dL). 

### 5.1. Renal

The Kaiser Permanente Northern California group analyzed >12,000 patients with four common cancers—breast, lung, bladder, and colon. They found that based on traditional GFR exclusion criteria of <60 mL/min, between 20 and 45% of patients would be excluded from clinical trials, with the highest impact observed in bladder cancer [44]. Harvey et al. showed that relaxing eligibility criteria with respect to renal function would allow >20% additional patients to be recruited in lung cancer trials [45]. Other studies in different cancers have shown similar findings with no increase in toxicity when appropriate dose adjustments were used for GFR [46,47,48,49]. In addition, novel agents such as immunotherapy and ADCs used in bladder cancer can be safely administered in patients with renal impairment, and arbitrary GFR cutoffs for eligibility for clinical trials in this setting are becoming less evidence-based [50,51]. 

### 5.2. Hepatic

Liver enzymes alone may not encompass the entire synthetic state of the liver and may not be sufficient to assess hepatic metabolism and drug tolerability [52,53]. A classic example is a patient with Gilbert Syndrome who may be wrongfully denied a clinical trial based on an asymptomatic bilirubin elevation. 

### 5.3. Cardiac

Although ejection fraction (EF) has been traditionally used as an indicator for myocardial function, the predictive value of baseline EF on the cardiotoxicity of cancer drugs is unclear [54]. We must consider whether a patient with an EF of 44% is different from one with an EF of 46%, if both are asymptomatic? 

Further, trials often mandate a pre-trial ECG to have a QTc < 450 ms. However, there is poor concordance between the different criteria used to measure corrected QTc [55], and asymptomatic ECG abnormalities do not clearly associate with cardiac events in phase 1 trials [56]. 

### 5.4. Other Laboratory Parameters

A similar example is the exclusion of a patient based on a trivial lab abnormality like a platelet count of 99 × 10^9^, when the study protocol defines eligibility of platelet count as ≥100 × 10^9^. 

In general, exclusion criteria with arbitrary and fixed cut-points are problematic. Until approximately 25 years ago, a study principal investigator was permitted to use clinical judgment regarding the appropriateness to include a patient who had a value close to an eligibility criterion cutoff point. The majority of these would classify as minor protocol deviations and would not need to be reported to IRB or compromise patient safety [57]. Regrettably, that latitude generally no longer exists. 

Other parameters, such as persistently elevated amylase, even in the face of normal lipase levels and normal pancreatic imaging, or elevated beta-HCG levels with normal pelvic ultrasound results, may reflect a paraneoplastic syndrome, rather than pancreatitis or pregnancy [58,59]; yet, registration trials often refuse to make pragmatic and common sense eligibility exceptions.

Laeeq et al. showed that even among phase 1 trials where drug toxicity is of particular concern, the use of restrictive criteria did not lead to lower dose-limiting toxicities, serious adverse events, or death with similar response rates in trials, irrespective of eligibility criteria [60]. Harvey et al. have demonstrated the near doubling of the number of eligible participants if criteria pertaining to brain metastasis, renal function, and prior malignancy are relaxed [45]. 

## 6. Other Subgroups of Interest

Older patients with an ECOG performance status (PS) of ≥ 2 are often excluded or underrepresented in clinical trials [61,62,63,64]. Although previous recommendations from various working groups have advocated for the inclusion of patients with an ECOG PS of 2 [65], the pharmaceutical industry has pushed back, citing inter-physician variability in the assessment of ECOG PS, with the potential for the inclusion of sicker patients, thereby jeopardizing the safety of study treatment [66]. 

Recent trials have shown the feasibility of administering drugs such as immunotherapy to older adults or patients with an ECOG PS of 2-3 with durable clinical benefit and without compromising safety [67,68]. While the absolute OS gain with an effective new therapy may be reduced in poor PS vs. good PS patients, the relative gain may be very similar [69]. Increasing the use of geriatric oncology specialists and assessments can play a substantial role in better assessing fitness and frailty for cancer treatment [70]. 

## 7. Summary and Recommendations—Trial Design and Eligibility (Table 1)

Taken together, current oncology trial eligibility criteria are overly restrictive, lack flexibility, and limit physician judgment. These criteria limit trial enrolment, the generalizability of study results, and adoption/access in real-world settings. Although some restrictions are necessary to define a study population and protect patient safety, there is a need to justify them with a scientific-based rationale. Ill-considered eligibility criteria unethically restrict access to novel and potentially beneficial therapies to patients. If patient safety is not compromised in the judgment of the investigator, patients should be offered the option of study participation. The disclosure of reasons for screen failures and their relevant supporting evidence published from study sponsors can also increase scientific rigor and the sense of transparency. 

Some recommendations to expand the eligibility criteria inclusivity of trials are listed in Table 1.

## 8. Section 2: Trial Activation (Table 2)

Activating a clinical trial can be a long and arduous process, taking lengthy time periods from when an institution receives a protocol until their first patient is recruited [71,72,73]. Previous studies have even found delays up to two years (with the longest being 5.2 years) for a trial to be activated [74]. A delay in trial activation has been associated with early trial closure due to poor recruitment, thus leading to a waste of money and resources [75,76]. Delays in the activation and completion of trials may lead to obsolete results if the SOC has changed. Additionally, most trials are carried out in patients with advanced disease, where such delays can translates to negative patient outcomes, satisfaction life-years lost, and a delay in accessing new effective treatments [77,78].

**Table 2:** Potential solutions to overcome delays in trial activation.

Problem	Solutions
Trial Activation Delay	Using a centralized ethics board like OCREB in Ontario [79,80,81]
	Development of broad protocols for central review with technical details only for IRBs [82,83,84,85]
	Multiple steps in parallel including submissions to various committees [72,86]
	Encourage communication between investigators, IRBs, and other stakeholders, e.g., the ReACT program in Canada [87,88]
	Activation of trials at smaller centers (with less hoops to go through) first, followed by larger centers

Several institutional-, regulatory-, and sponsor-related hurdles need to be overcome before a trial can be activated [89]. Dilts et al. found that a minimum of 296 distinct steps were involved in activating an NCI CTEP trial and receiving Institutional Review Board (IRB) approval. These involved 21 loops (i.e., points where the protocol would be returned to a previous point for changes) and 11 major stopping points [74]. Recent work by Williams et al. at a tertiary center in the US found a median activation time of 182 days but 21 potential bottlenecks causing delays in trial activation [72]. Most of these steps are conducted sequentially, compounding delays. Notably, more stringent regulations do not correlate with improved patient safety, even in early phase trials, where the toxicity profile is uncertain to a large extent [90,91]. 

The healthcare system works with constrained budgets, resources, and workforce capacity. Ever increasing research costs and more complex trial designs can lead to lengthy inter-departmental approvals and budget negotiations. Notwithstanding these challenges, institutions and trial sponsors need to evaluate innovative processes to ensure efficiency, capacity, and sustainability across clinical research portfolios. We discuss some possible solutions in Table 2.

## 9. Section 3: Trial Conduct (Table 3)

Roadblocks during the conduct of a clinical trial create unnecessary clinic visits, tests, paperwork, and overall burden to patients and staff. This contributes to time toxicity and costs [92], even being so cumbersome that a research center is unable to activate or continue trial activity. For example, some trials mandate scans in a certain time interval prior to enrollment. A lung cancer patient with appropriate staging investigations may have to undergo repeat scans to fit mandated trial timelines. This can lead to a delay in starting treatment and may not provide any additional clinically relevant information. Some trials mandate follow-up schedules that are vastly more intensive than routine clinical practice (bloodwork, scans, and appointments), posing enormous unnecessary burdens on the patient and trial team (physicians [oncologists, radiologists, etc.], nurses, and support staff). Occasionally, non-trial patients are impacted so that mandated additional trial patient assessments can be accommodated. Inconsequential (to patient or data safety) protocol deviations ensue, causing additional work. Moreover, these practices perpetuate inequity, as patients living far from large centers have impeded access to trials due to practicality and transportation issues.

**Table 3:** Issues with trial conduct and potential solutions.

Trial Conduct Issues	Potential Solutions
Cross-over delays with mandatory central confirmation prior to cross-over	Investigator assessment of scans should be enough if progression is equivocal and investigator feels a change of treatment is warranted. Mandatory central confirmation should be removed [93]
Mandating central confirmation of lab tests for which standardized assays are available	Should be removed when tests are available as standard of care
No. of visits (both baseline and follow up)—adds to “time toxicity” of cancer care	Efforts should be made to minimize trial visits to save both patient and physician time [94,95,96]. Additional measures include the following: Use of tele-health for follow-up Use of e-consent for pre-trial screening and consent Home visits by trial nurses for side effect monitoring/PRO assessment
Time spent at each visit with no. of procedures—exams, labs, ECGs, etc.	Minimize procedures that do not add clinical value to the trial. This must be carried out at the level of design and protocol development in concert with the sponsor
Mandating investigations at the cancer center itself rather than close to home	Decentralization with allowing routine tests and imaging at centers close to the patient’s home with imaging reviews by hospital radiologists [96,97,98] Flexibility with imaging assessments based on investigator judgment
Drug pickups mandated at hospital pharmacies	Shipping drugs to patients’ pharmacy or directly to patient
Dose adjustment regulations on trial for responding patients	Having an “investigator judgment” dose level, which should be recorded and reported rather than rigid rules for taking responding patients off trial for dose reductions

Even post-pandemic, most trials do not allow the use of tele-health for follow-up assessments when it is routinely used in clinical care. Available evidence suggests that tele-health based follow-up is safe, accepted by patients and oncologists, and is also cost-effective [99,100,101,102]. Several trials insist on in-person assessments for collection of safety, follow-up, and PRO data, even after patients have progressed in the trial and have gone onto another treatment even though they might live far out from the cancer center. Whether strict regulations such as these improve patient outcomes is not well supported by evidence, but intuitively seems unnecessary and burdensome [77].

Certain procedures are mandated by trials, and likely these are far too many. A review of phase 1 trials showed that a mean of 3.16 physician exams, 5.6 vitals sign measurements, 4.36 ECGs, 18 non-pharmacological blood draws, and 15.1 pharmacological blood draws were carried out in just the first 28 days of a phase 1 trial [103]. Although a higher degree of scrutiny for phase 1 trials can be justified given the experimental nature of treatments, even this may be excessive [56]. In 2010, excessively stringent clinical research regulation cost an estimated 2.7 million dollars per life-year saved. This drives up the research costs, which only the large pharmaceutical companies can afford to pay, and this in turn drives the focus away from investigator-initiated and cooperative group studies, which often answer more patient-centric questions [77]. 

Many studies mandate the central confirmation of immunohistochemical and genomic markers, even though validated and standardized assays for such biomarkers exist. Examples include the requirement of the central confirmation of PD-L1 testing in NSCLC, the confirmation of genomic biomarkers for targeted therapy trials in oncogene-addicted NSCLC, confirmation of HER-2 IHC in breast cancer, etc. When local accredited laboratories perform these tests, mandating central confirmation can only delay and restrict access, without a clear benefit. After the conclusion of a central trial, hospitals will not start sending specimens for central confirmation, they will continue to rely on validated local testing. 

Cross-over is allowed in some trials, where patients on control arm therapy can access the experimental treatment at progression. However, when radiologic PFS is a primary endpoint, the central confirmation of disease progression may be required prior to cross-over, even if clear clinical progression is evident, as judged by the treating investigator. This may be contrary to Good Clinical Practice (GCP) by compromising patient outcomes when treatment is delayed/denied due to mandated protocol procedures. If patients withdraw from such a study prior to the completion of the central review, there is a loss of important data due to censoring.

Trials can also impose restrictions on the timing of drug delivery (e.g., with 1–2 days of due date) and drug administration times between preparation in the pharmacy and administration to the patient. This poses unnecessary pressure on often already overburdened day care units and pharmacies and can impact the care of non-trial patients. While there may be good scientific reasons to recommend the accurate timing of drug dosing, pragmatic considerations could be adopted.

We have summarized some suggestions to make trial conduct more patient-centric in Table 3.

## 10. Conclusions

A number of examples discussed above go against the principle of common sense oncology, for which there has been a lot of recent advocacy [104]. The examples discussed in this manuscript are reflective of situations that the authors have encountered and bring a desire for improvement in patient care in the context of clinical trials. Some of the recommendations have been highlighted before, and if implemented, would certainly help in ‘cutting out the stupid’ that plagues oncology clinical trials. The development of choosing wisely choosing recommendations for clinical research might be a step worth considering, given the success of similar recommendations for clinical practice [105]. 

Similar issues were noted during the early days of AIDS research and a change in mindset and approach from the community was the key to eventual rapid progress. While these recommendations may seem relatively straightforward to implement, a more balanced and interactive collaboration between stakeholders (sponsors, patients, investigators, institutions, and regulators) is required. Patients should be part of clinical trial design and steering committees to advocate for patient interests and priorities, rather than satisfying only commercial interests. Ultimately, pragmatic and inclusive clinical trial reform may lead to commercial benefits with faster and cheaper clinical trials. If we achieve that balance, many of the procedures that are currently ingrained in clinical trial design, enrollment, activation, and conduct would eventually be taken care of, therefore ‘cutting out the stupid’. 

A recent example of a pragmatic clinical trial is the SWOG 2302 (PRAGMATICA LUNG) testing ramucirumab plus pembrolizumab vs. standard of care (as determined by investigators) in the second line treatment of NSCLC [106]. The trial’s aims were to remove traditional barriers to eligibility, as discussed in this paper. The trial included patients with an ECOG performance status of 2, did not mandate strict timing for lab tests and scans, permitted investigator-assessed PFS as opposed to central assessment, used OS as the primary end point, and required mandatory reporting of only serious adverse events, thus minimizing paperwork [107]. The success of this trial might pave the way for more patient-centered studies and help in establishing the feasibility of such studies.

George Bernard Shaw wrote ‘Progress is impossible without change, and those who cannot change their minds cannot change anything’. We hope that this review will highlight the need for change in clinical trial conduct to ensure progress for people with cancer.
